# C-C Motif Ligand 20 (CCL20) and C-C Motif Chemokine Receptor 6 (CCR6) in Human Peripheral Blood Mononuclear Cells: Dysregulated in Ulcerative Colitis and a Potential Role for CCL20 in IL-1β Release

**DOI:** 10.3390/ijms19103257

**Published:** 2018-10-20

**Authors:** Helene Kolstad Skovdahl, Jan Kristian Damås, Atle van Beelen Granlund, Ann Elisabet Østvik, Berit Doseth, Torunn Bruland, Tom Eirik Mollnes, Arne Kristian Sandvik

**Affiliations:** 1 Centre of Molecular Inflammation Research, Norwegian University of Science and Technology (NTNU), 7030 Trondheim, Norway; helene.k.skovdahl@ntnu.no (H.K.S.); jan.k.damas@ntnu.no (J.K.D.); atle.granlund@ntnu.no (A.v.B.G.); ann.e.ostvik@ntnu.no (A.E.Ø.); berit.doseth@ntnu.no (B.D.); t.e.mollnes@medisin.uio.no (T.E.M.); 2 Department of Clinical and Molecular Medicine, NTNU, 7030 Trondheim, Norway; torunn.bruland@ntnu.no; 3 Department of Infectious Diseases, St. Olav’s University Hospital, 7030 Trondheim, Norway; 4 Department of Gastroenterology and Hepatology, St. Olav’s University Hospital, 7030 Trondheim, Norway; 5 Clinic of Medicine, St. Olav’s University Hospital, 7030 Trondheim, Norway; 6 Department of Immunology, Oslo University Hospital and University of Oslo, 0372 Oslo, Norway; 7 Research Laboratory, Department of Laboratory Medicine, Nordland Hospital, 8005 Bodo, Norway; 8 K.G. Jebsen TREC, University of Tromsø, 9037 Tromsø, Norway

**Keywords:** inflammatory bowel disease, CCL20, CCR6, peripheral blood mononuclear cells, interleukin-1β

## Abstract

The chemokine C-C motif ligand 20 (CCL20) is increased in the colonic mucosa during active inflammatory bowel disease (IBD) and can be found both in the epithelium and immune cells in the lamina propria. The present study investigated CCL20 and C-C motif Chemokine Receptor 6 (CCR6) in peripheral blood mononuclear cells (PBMCs) (*n* = 40) from IBD patients and healthy controls, to identify inductors of CCL20 release encountered in a local proinflammatory environment. CCL20 release from PBMCs was increased when activating TLR2/1 or NOD2, suggesting that CCL20 is part of a first line response to danger-associated molecular patterns also in immune cells. Overall, ulcerative colitis (UC) had a significantly stronger CCL20 release than Crohn’s disease (CD) (+242%, *p* < 0.01), indicating that the CCL20-CCR6 axis may be more involved in UC. The CCL20 receptor CCR6 is essential for the chemotactic function of CCL20. UC with active inflammation had significantly decreased *CCR6* expression and a reduction in CCR6^+^ cells in circulation, indicating chemoattraction of CCR6^+^ cells from circulation towards peripheral tissues. We further examined CCL20 induced release of cytokines from PBMCs. Stimulation with CCL20 combined with TNF increased IL-1β release from PBMCs. By attracting additional immune cells, as well as inducing proinflammatory IL-1β release from immune cells, CCL20 may protract the inflammatory response in ulcerative colitis.

## 1. Introduction

Inflammatory bowel disease (IBD) includes ulcerative colitis (UC) and Crohn’s disease (CD), which share a disease pattern of intermittent inflammation of the gastrointestinal mucosa. The general view of the diseases is that inflammation occurs through an aberrant immune reaction to a normally tolerated gut flora in genetically susceptible individuals [1]. While many factors involved in driving a chronic inflammatory response have been identified, it is hitherto unknown what initiates the inflammation in IBD. The gastrointestinal mucosa has been established as an important constituent of the immune system due to its close contact with the microbiome [2] and the number of immune cells within the mucosa is higher in the gut than in other organs [3]. The balance between inflammatory response and tolerance towards gut flora depends on the proportions of regulatory and effector immune cells in the mucosa.

Chemokines participate in the regulation of the immune cell composition, since cells expressing the corresponding receptor are directed towards the site of chemokine production (e.g., mucosal cells), along a concentration gradient [4,5,6]. CCL20 is a C-C motif chemokine ligand, which has C-C motif Chemokine Receptor 6 (CCR6) as its sole receptor [7,8] and tissues producing CCL20 recruit CCR6 expressing immune cells [9,10,11,12]. As CCL20 recruits both regulatory T cells and Th17 cells, a change in CCL20 expression can potentially alter the balance between proinflammatory and regulatory responses [13,14,15]. CCL20 is increased in the mucosa of IBD patients [16,17,18] and CCL20 and CCR6 have been identified as IBD susceptibility genes and are suggested as treatment targets in inflammatory conditions [19,20]. Studies on CCL20 and CCR6 in mononuclear cells from circulation of patients with UC indicate that CCL20 mRNA is upregulated during active disease compared to healthy controls [21,22,23]. Cayaette et al. proposed serum CCL20 as a potential biomarker for active CD [24]. We have previously found CCL20 expression in both the epithelium and in mononuclear cells in the lamina propria [18].

The activity of circulating immune cells is of interest during active inflammation. Indeed, the current hypothesis on the extraintestinal manifestations in IBD is that immune cells activated in the intestinal mucosa migrate to other organs and initiate inflammation [25,26]. Mesko et al. identified expression patterns specific for IBD and rheumatoid arthritis in PBMCs, suggesting that new insights may be gained from studying patient mononuclear cells in inflammatory diseases [27]. The current understanding is that CCL20 is released mainly in response to other inflammatory cytokines, such as TNF and IL-1β, indicating a role for CCL20 when inflammation is ongoing. However, the mechanisms behind CCL20 release from immune cells in IBD have not been thoroughly investigated.

The aim of this study was to investigate CCL20 and CCR6 expression by immune cells during IBD and clarify if CCL20 release from immune cells can be a primary response to luminal components. In addition, we explore immune cell cytokine responses to CCL20 stimulation.

## 2. Results

### 2.1. Patient Characteristics

Peripheral blood mononuclear cells (PBMCs) from 40 subjects in an established IBD cross sectional study biobank [28] were included in this work (Healthy controls (N) = 8, UC = 16, CD = 16). Patient characteristics are summarized in Table 1, where patients with inactive UC (Mayo Score) or CD (Harvey Bradshaw Index) all had clinical scores of 0. Seven healthy controls were also included. Patients using azathioprine, TNF inhibitors or other immunomodulators were excluded. Use of 5-ASA/S-ASA was more common in UC compared to CD. Two subjects had extraintestinal manifestations (ankylosing spondylitis (*n* = 1) and primary sclerosing cholangitis (*n* = 1)).

### 2.2. TLR2/1, NOD2 and IL-1β Strongly Induce CCL20 in PBMCs

**Figure 1:**
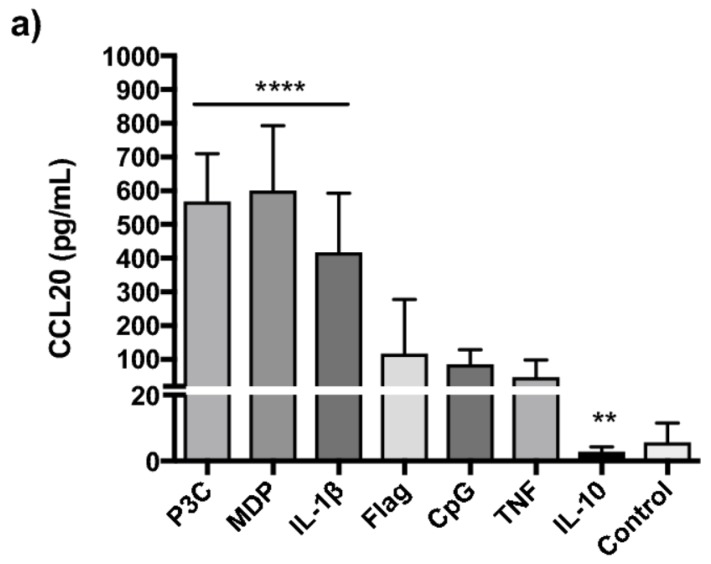

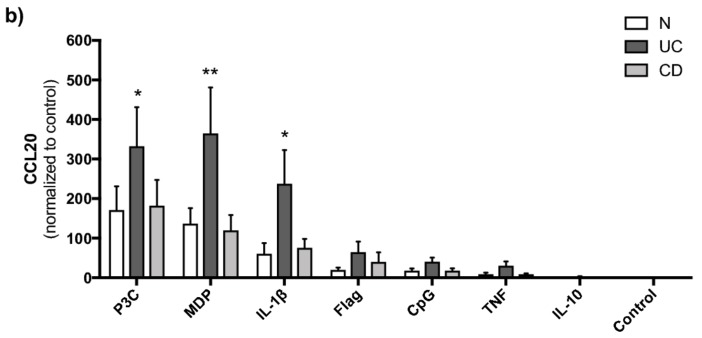
C-C motif ligand 20 (CCL20) release from PBMCs CCL20 (pg/mL) release from PBMCs (*n* = 40), following stimulation with lipopeptide Pam3CysSK4 (P3C) (TLR1/2), peptidoglycan component muramyl dipeptide (MDP) (NOD2), unmethylated CpG dinucleotides (CpG) (TLR9), flagellin (TLR5), interleukin (IL) 1β (IL-1β), IL-10 and tumour necrosis factor (TNF). (**a**) CCL20 (pg/mL) release from PBMCs (*n* = 40), plotted as median with 95%CI. Statistical comparison was performed using Wilcoxon matched-pairs signed rank test with levels of significance denoted by **** *p* < 0.0001 versus control, ** *p* < 0.01 versus control. (**b**) CCL20 release following stimulation normalized to release in control conditions, in PBMCs from healthy controls (N) (*n* = 8), ulcerative colitis (UC) (*n* = 16) and Crohn’s disease (CD) (*n* = 16) patients. Mean with SEM are plotted. Statistical comparison was performed using unpaired *t*-test with levels of significance denoted by * UC vs. CD *p* < 0.05, ** UC vs. CD *p* < 0.01.

Since mechanisms for CCL20 release from PBMCs have not been thoroughly investigated, we first stimulated PBMC from four healthy individuals in a pilot study with a broad panel of ligands as described in Section 4.7. This screening revealed that TLR2/6, TLR3, TLR4 and TLR7/8 yielded minimal CCL20 release (<200 pg/mL) (Supplementary Figure S1). Consequently, we excluded these ligands in the following stimulation assay in 40 subjects. Cell survival after thawing was generally high (mean 93 ± 3.3%). Spontaneous CCL20 release from PBMCs was minor and there were no group differences. In general, P3C (+633%, *p* < 0.0001), MDP (+633%, *p* < 0.0001) and IL-1β (+359%, *p* < 0.0001) yielded the strongest CCL20 responses, while IL-10 reduced release (−63%, *p* < 0.01) compared to unstimulated control cells (Figure 1a). IL-1β release following activation of TLR2/1 and NOD2 is established. We therefore investigated whether CCL20 release after P3C and MDP stimulation was secondary to IL-1β release. IL-1β neutralization prior to IL-1β stimulation reduced both CCL20 (−54% ± 10, *p* = 0.006) and IL-6 (−84% ± 10) levels to that seen in unstimulated isotype controls. NOD2 mediated release was mildly affected (−33% ± 12, *p* = 0.047) while TLR2/1 mediated release was unaffected (*p* = 0.36) (Supplementary Figure S2). To exclude freeze-thaw bias, the experiments were repeated in freshly isolated PBMCs (Supplementary Figure S3) which yielded similar responses. UC subjects released more CCL20 than CD subjects upon stimulation (Figure 1b), relative to spontaneous release from the PBMCs. This was statistically significant after P3C, MDP and IL-1β stimulation and also when CCL20 response was pooled for all stimuli (+242%, *p* < 0.01).

### 2.3. CCL20 Can Increase TNF Induced IL-1β Release

**Figure 2:**
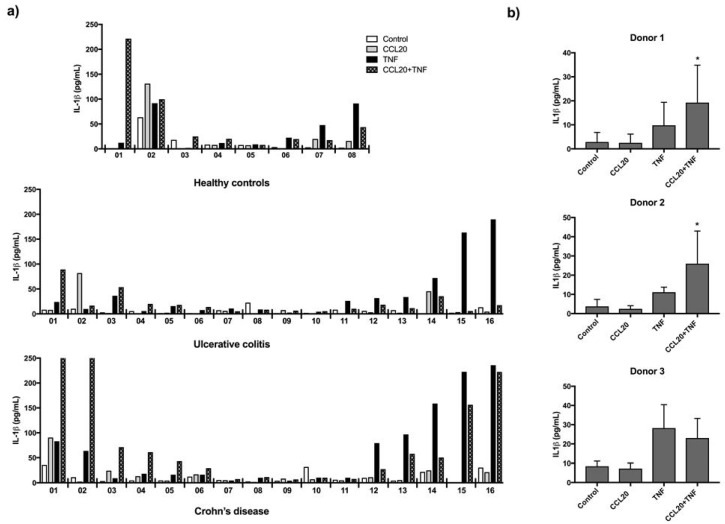
IL-1β response to CCL20 stimulation in PBMC. IL-1β release (pg/mL) from PBMCs after stimulation with CCL20 alone or in combination with TNF (CCL20+TNF), TNF alone, or no stimuli (Control). (**a**) Assay in PBMCs from 40 individuals. Individual values are plotted. (**b**) IL-1β released from PBMCs from three healthy donors in three stimulation assays, plotted as mean with 95% CI. Statistical comparison was performed using unpaired *t*-test with levels of significance denoted by * *p* < 0.05, CCL20 ^+^ TNF versus TNF.

A pilot CCL20 stimulation assay indicated that CCL20 in combination with TNF could induce release of IL-1β and therefore PBMCs from 40 subjects were stimulated with CCL20 in combination with TNF and the release of IL-1β measured. Cell survival after thawing was high (mean 93 ± 3.3%). There were no group differences in response to CCL20 alone or in combination with TNF. However, some individuals showed a strong response to CCL20 stimulation alone and/or in combination with TNF (Figure 2a). Similarly, the TNF response was strong in a subset of subjects, while barely present in others. Variation between individuals is common and can be of clinical significance in patient-derived samples. To confirm that variation reflects biological differences, we investigated technical variation in PBMC assays. PBMCs from three healthy individuals were isolated and stored in multiple aliquots on liquid nitrogen. Three separate stimulation assays were conducted on frozen aliquots of PBMCs in all three donors (Figure 2b). Cell viability after thawing was generally high (mean 92 ± 2.5%). In donor 1 and 2, IL-1β concentration in supernatant was highest after stimulation with CCL20 combined with TNF in all three assays (versus TNF stimulation alone, *p* = 0.0469 in donor 1 and *p* = 0.0475 in donor 2) while in donor 3 TNF alone repeatedly yielded the highest IL-1β levels in supernatant with no increase when CCL20 was used as costimuli (Figure 2b). The results suggest that CCL20 can increase IL-1β release in combination with TNF but there are important inter-individual differences.

### 2.4. CCR6 Gene Expression Is Downregulated in PBMCs from Active Ulcerative Colitis

**Figure 3:**
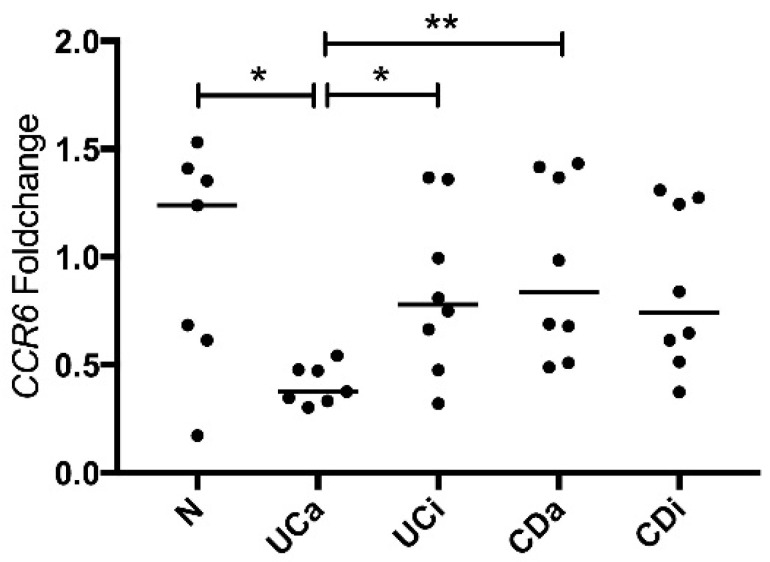
CCR6 mRNA PBMCs. CCR6 mRNA in PBMCs (*n* = 38) from healthy controls (N), active ulcerative colitis (UCa) inactive ulcerative colitis (UCi), active Crohn’s disease (CDa) and inactive Crohn’s disease (CDi). Fold changes were determined relative to mean expression of the reference genes *beta actin*, *TATA binding protein* and *eukaryote 18s rRNA* and to the mean expression level in N. Individual fold change and median are plotted. Statistical comparison was performed using Kruskal-Wallis test followed by Mann-Whitney test with levels of significance denoted by * *p* < 0.05 and ** *p* < 0.01.

To investigate *CCL20* and *CCR6* expression in circulating immune cells, we measured mRNA by qRT-PCR in PBMCs from N (*n* = 7), UCa (*n* = 7), UCi (*n* = 8), CDa (*n* = 8) and CDi (*n* = 8). There were no differences in CCL20 mRNA levels between the disease groups (Figure S4). CCR6 mRNA in PBMCs was significantly lower in UCa compared to N, UCi and CDa (Figure 3). No such difference was seen for the CD groups. To investigate whether high levels of CCL20, as is found in the colonic mucosa during UCa [17,18], can lead to a downregulation of CCR6 in immune cells, we measured CCR6 mRNA in PBMCs after stimulation with CCL20. PBMCs from three healthy individuals stimulated with CCL20 for 20 h, showed no change in CCR6 mRNA compared to unstimulated control cells (mean CCR6 mRNA in control condition: 0.76 ± 0.24, mean CCR6 mRNA in CCL20 stimulated PBMCs: 0.81 ± 0.31. *p* = 0.54).

### 2.5. CCR6^+^CD4^+^ Cells among PBMCs Are Decreased in Active Ulcerative Colitis

**Figure 4:**
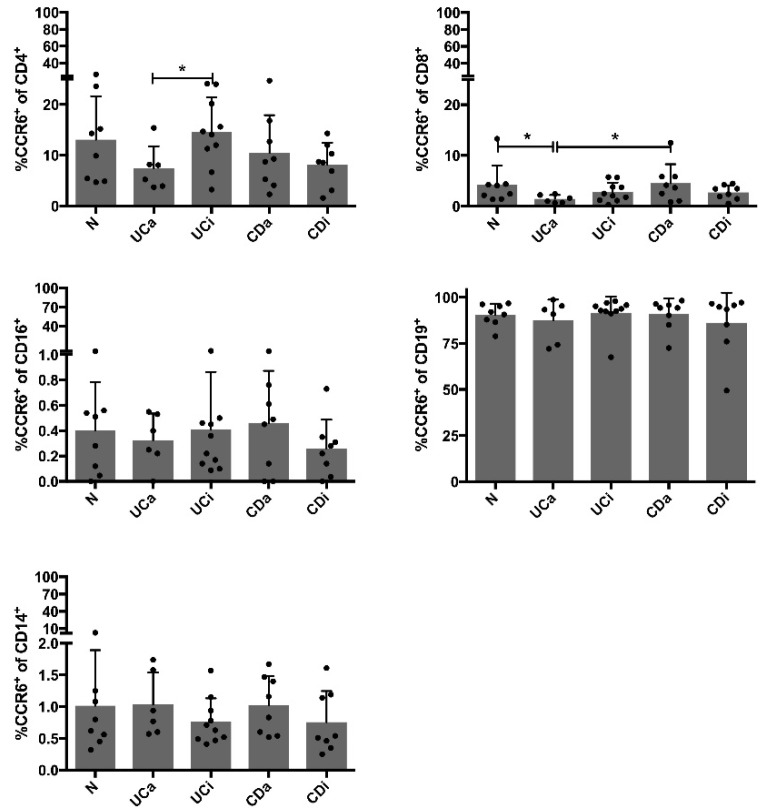
CCR6^+^ populations in PBMCs Flow cytometry measuring CCR6^+^ cells in the main cell populations (CD4^+^ T cells, CD8^+^ T cells, CD16^+^ NK cells and CD19^+^ B cells and CD14^+^ monocytes), in 10,000 PBMCs from each subject (*n* = 40), in the groups healthy controls (N), active ulcerative colitis (UCa) inactive ulcerative colitis (UCi), active Crohn’s disease (CDa) and inactive Crohn’s disease (CDi). Bars show mean frequency (%) with standard deviation of CCR6^+^ cells in different cell populations and individual values are plotted. Statistical comparison was performed using unpaired *t*-test with levels of significance denoted by * *p* < 0.05.

*CCR6* gene expression results lead us to further investigate surface protein expression of CCR6 in PBMCs by flow cytometry (Figure S5). Main cell populations were similar in disease groups (Table S1 and Figure S6). CD19^+^ cells and CD4^+^ cells provided the largest populations of CCR6^+^ cells (Figure 4). CCR6 was expressed on 89.5 ± 10.4% of CD19^+^ cells and on 11.1 ± 6.9% of CD4^+^ cells and total frequency within lymphocytes is 45.9 ± 10.3 for CD4^+^ cells and 5.3 ± 1.7% for CD19^+^ cells. There were no significant differences between disease groups in expression of CCR6 on CD19^+^ cells. However, there were fewer CCR6^+^CD4^+^ cells in UCa compared UCi (Figure 4). Furthermore, the frequency of CCR6^+^CD8^+^ cells was significantly lower in UCa compared to N and CDa (Figure 4). These findings indicate that the vast majority of CD19^+^ cells expressed CCR6 and this proportion was not altered during disease, while peripheral blood CCR6-expressing CD4^+^ and CD8^+^ cells were decreased during UCa. Thus, both *CCR6* mRNA and expression of surface CCR6 receptor (protein) on PBMCs are reduced in UCa. This combined with stronger CCL20 release from UC subjects compared to CD subjects, suggests that the CCL20-CCR6 axis is triggered more in UC than in CD.

## 3. Discussion

Our previous studies, using both immunohistochemistry and in situ hybridization showed a high density of CCL20 positive immune cells in the lamina propria during active IBD [18]. In the present study PBMCs had low basal CCL20 gene expression and release with no difference between disease groups, suggesting that the regulation of CCL20 expression in immune cells mainly occurs after immune cells have reached the colonic mucosa. CCL20 was highly inducible in PBMCs, in particular through TLR2/1, NOD2 and IL-1β. NOD2 induced release of CCL20 is supported by Hausmann et al., who showed that CCL20 release from macrophages is NOD dependent [29]. We showed for the first time that PBMCs from the UC group tend to have a stronger NOD2, TLR2/1 and IL-1β induced CCL20 release than PBMCs from the CD group, suggesting a disease dependent difference in CCL20 involvement. Response to IL-1β stimulation and reduced release following IL-10 stimulation indicated that CCL20 release from PBMCs is a trait of a proinflammatory environment. We proceeded to show that CCL20 release from PBMCs after TLR2/1 and NOD2 activation is not only secondary to IL-1β production but also a direct response to stimulation of these PRRs. It has been established that CCL20 has antimicrobial actions and release of CCL20 in response to bacterial components could be part of an antibacterial response, both directly and through immune cell recruitment. Cells recruited to the mucosa along a CCL20 gradient may commence CCL20 production upon arrival, further accelerating cell recruitment, a process common in chemokine biology [30].

In the colonic mucosa, *CCR6* is increased during active IBD and in our previous study [18] we found an increase in CCR6 mRNA positive immune cells in lamina propria in IBD during active inflammation, compared to IBD in remission and healthy controls. As the main recognized function of CCL20 is recruitment of CCR6^+^ immune cells, investigating the expression of CCR6 on immune cells is relevant. We found reduced CCR6 mRNA and CCR6^+^ cells in PBMCs from active UC compared to inactive UC, active CD and healthy controls. Lee et al. have shown that frequency of circulating CCR6^+^ cells is reduced during active UC and suggested that CCR6^+^ cells migrate from the circulation to the inflamed bowel mucosa and are thus reduced in peripheral blood [21]. An alternative explanation is downregulation of *CCR6* in response to an increase in CCL20 production in the mucosa. The expression of chemokine receptors is readily turned on and off through stages of maturation and activation [12,31,32], allowing traffic between and within tissues. However, we found no effect of CCL20 stimulation on *CCR6* expression in PBMCs, indicating that migration is a likely explanation.

We found novel differences between UC and CD for CCL20 release and in CCR6 expression in PBMCs. As the pair is responsible for recruitment of both Treg and Th17 cells, recognised as important factors in IBD [33], this is an interesting discrepancy. Skewed sampling may lead to systematic group differences, for example, if the UC group tended to be more diseased than the CD group. However, there were no indications that this was the case in our patient material. Both groups consisted of individuals with active disease and individuals in clinical and microscopic remission. Our findings indicate that treatment targeting the CCR6 receptor for IBD could be most effective in UC. Recently, blocking CCR6 was tested in a murine model of psoriasis and resulted in a reduced recruitment of Th17 cells to the plaque and reduced plaque thickness [34].

There are high levels of both CCL20 and TNF in the colonic mucosa during active inflammation. These studies were done using PBMCs rather than immune cell subtypes, since in vivo immune cells interact and responses may occur due to these interactions while single cell populations may not respond alone. Our results suggest a new role for CCL20 as a costimulus during TNF-mediated cytokine release from PBMCs. CCL20 has previously been shown to increase release of IL-6 in chondrocytes [35] but CCL20 induced release of IL-1β from immune cells is a novel observation. In the 40 individuals, there were responders and non-responders. Flow cytometry showed individual variation in cell population frequencies but there are no consistent correlations with responsiveness to CCL20 (Supplementary Data file). We tested reproducibility of PBMC responses and found that the responsiveness to CCL20 combined with TNF is maintained within the individuals PBMCs. The three donors were blood donors and considered healthy but subclinical conditions which might affect immune cell activity cannot be dismissed. Inter-individual variation in response to IBD treatment is known to be large and how this might correlate to variability in responses in ex vivo models such as PBMCs is an interesting focus for future investigations.

Previous studies have suggested PBMC *CCL20* gene expression as a surrogate marker of disease activity in IBD [21,22,36]. We found no difference in CCL20 mRNA in PBMCs between the disease groups. There are some known differences in genetic risk factors between IBD patients in Asia and Europe [19]. Choi et al. detected SNPs in the promoter region of the *CCL20* gene in a Korean population [36] and the presence of such variants could differ between populations. Lee et al. found that 5-ASA and dexamethasone influenced CCL20 expression in PBMCs [21]. In our sample set, most UC subjects used 5-ASA, which might explain why CCL20 mRNA levels were not increased compared to healthy control subjects. However, few CD subjects used 5-ASA and the CCL20 expression in PBMCs from CD is not significantly different from healthy controls or UC.

CCL20 is expressed by epithelium during homeostasis and inflammation and exploring the CCL20-CCR6 controlled interplay between intestinal epithelial cells and immune cells will expand the understanding of the chemokine-chemokine receptor axis during health and disease. In this study, we further explore a role for CCL20 in IBD pathogenesis and propose that this chemokine and its receptor CCR6 are of particular interest in UC. CCL20 might indeed be a response to detection of danger-associated molecular patterns, as CCL20 is released as a response to PRR activation and recruits immune cells. Upon stimulation with CCL20 and TNF immune cells can respond with IL-1β release. By attracting additional immune cells, as well as inducing proinflammatory IL-1β release from immune cells, CCL20 may protract the inflammatory response.

## 4. Materials and Methods

### 4.1. Patient Material

Study material was randomly selected from an IBD cross sectional study biobank [28], at the Department of Gastroenterology and Hepatology, St. Olav’s University Hospital. The biobank includes clinical information, blood fractions including PBMCs and tissue samples from healthy controls (N), patients with active (UCa) or inactive (UCi) ulcerative colitis; or active (CDa) or inactive (CDi) Crohn’s disease. Healthy controls (N) were recruited among patients with gastrointestinal symptoms where standard diagnostic procedures revealed no significant disease. Patients were classified based on clinical, endoscopic and histological evaluation. Pinch biopsies were collected from endoscopically assessed maximally inflamed colonic mucosa, or from the hepatic flexure when there was no inflammation. Haematoxylin and eosin stained sections were classified as “normal,” “chronic inflammation” or “chronic active inflammation” based on neutrophil and mononuclear cell infiltration. Patients using azathioprine or TNF inhibitors were excluded. Peripheral blood mononuclear cells (PBMCs) from 43 subjects were included in this study (N = 9, UCa = 8, UCi = 10, CDa = 8 and CDi = 8) (Table 1) stratified using Mayo score for UC and Harvey-Bradshaw index for CD. Seven healthy control individuals were also included.

### 4.2. Ethical Considerations

The study was approved (19 April 2013) by the Regional Medical Ethics Committee (Reference number 5.2007.910 and 2013/212/REKmidt). All subjects included in the study gave informed written consent. All experiments were performed in accordance with relevant guidelines and regulations.

### 4.3. Sample Preparation

Peripheral blood mononuclear cells (PBMCs) were isolated from heparinized venous blood by centrifugation (800× *g* for 20 min at room temperature) on Lymphoprep mononuclear cell separation medium (Axis-Shield, Oslo, Norway). PBMCs were stored on liquid nitrogen in medium with human AB^+^ serum and DMSO 10%. To study mechanisms of CCL20 release, PBMCs were isolated from buffy coat from healthy blood donors at the Department of Immunology and Transfusion Medicine, St. Olav’s University Hospital.

### 4.4. Gene Expression Analyses for CCL20 and CCR6 in PBMCs

PBMCs from 40 subjects were thawed in medium consisting of 1640 RPMI with glucose and L-glutamine (Life Technologies, Paisley, UK), augmented with 5% human AB^+^ serum, 2 mM glutamine and 0.05% gentamicin (supplemented RPMI). Cells were washed with PBS, followed by centrifugation (8 min at 450× *g*). The cell pellet was snap frozen in liquid nitrogen and kept at −80 °C. PBMCs from three healthy donors stimulated with CCL20 and unstimulated control cells were washed with PBS and snap frozen in liquid nitrogen, after lysis buffer treatment. RNA extraction was done in lysis buffer using the RNeasy mini kit and QiaShredder columns from Qiagen (Hilden, Germany). RNA quality control was done with NanoDrop Spectrophotometer (Thermo Fisher Scientific, Waltham, MA, USA) for quantity and purity. Qbit (Thermo Fisher Scientific, Waltham, MA, USA) and Bioanalyzer (Agilent Technologies, Santa Clara, CA, USA) were used to determine RNA integrity. Samples with a RIN < 4.5 were excluded from the final analysis (final *n* = 38). Isolated RNA was stored in cryotubes in liquid nitrogen. Reverse transcription was done using the High Capacity RNA-to-cDNA kit (Applied Biosystems, Foster City, CA, USA) according to the manufacturer’s instructions. PerfeCTa assay was used for CCL20 and CCR6 detection with PerfeCTa qPCR FastMix (Quantabio, Beverly, MA, USA) and TaqMan probes (*CCL20*: probe ID Hs01011368_m1, *CCR6*: probe ID Hs01890706_s1, (Applied Biosystems, Foster City, CA, USA) with the reference genes *beta actin* (ACTB: probe ID Hs01060665_g1), *TATA box binding protein* (TBP: probe ID Hs00427620_m1) and *eukaryotic 18S* rRNA (18S: probe ID Hs99999901_s1). StepOnePlus Real-Time PCR System and StepOne software v. 2.1 (Applied Biosystems, Foster City, CA, USA) was used for all PCR procedures. Fold changes were calculated using the ΔΔ*C*_T_ method, where individual expression levels were relative to the mean of reference genes and further relative to the mean in the healthy control group (N).

### 4.5. Flow Cytometry

Flow cytometry analysis was performed to identify PBMC subpopulations and surface expression of CCR6. One million cells from each of the 40 subjects were incubated with human Fc Receptor binding inhibitor from eBioscience (Thermo Fisher Scientific, Waltham, MA, USA) for 20 min. Cell were further stained for 30 min at room temperature with the following anti-human antibodies: CD8a PerCP (HIT8a), CD14 Pacific Blue (HCD14) and CD19 APC (HIB19) from BioLegend (San Diego, CA, USA), CD4 FITC (RPA-T4) from eBioscience (Thermo Fisher Scientific, Waltham, MA, USA) and CCR6 PE (11A9) or isotype control IgG1κ PE (MOPC-21) and CD16 APC-H7 (3G8) from BD Bioscience (Franklin Lakes, NJ, USA). After incubation with fix/lysis buffer from eBioscience (Thermo Fisher Scientific, Waltham, MA, USA) for 10 min at room temperature, cells were washed twice in FACS buffer (PBS with 0.1% BSA) and resuspended in PBS. Compensation controls were prepared using Anti-mouse Igκ/Negative Control Compensation Particle Set from BD Bioscience (Franklin Lakes, NJ, USA) according to the manufacturer’s recommendations. Flow cytometry was performed on a BD FACS Canto II flow cytometer with FACS Diva software from BD Bioscience (Franklin Lakes, NJ, USA) (10,000 events per sample) and samples were analysed using FlowJo v10 software from Flow Jo, LLC (Ashland, OR, USA). 

### 4.6. PBMC Stimulation Assays

For all assays in PBMCs, cells were carefully thawed and incubated in supplemented RPMI and cell count and viability was measured on Countess Automated Cell Counter (Life Technologies, Grand Island, NY, USA). Cells were seeded in 96-well plates, 0.5 million cells per well in every assay and incubated at 37 °C, 5% CO_2_. All supernatants were stored at −80 °C. Unstimulated cells served as controls in all assays. As mechanisms for CCL20 release from PBMCs have not been thoroughly investigated, we performed a pilot study to select putative ligands. PBMC from four healthy individuals were stimulated with ligands to toll-like receptor (TLR) 2/1 (pam3cysSK4-P3C), TLR2/6 (lipomannan-LM), TLR3 (Poly IC), TLR4 (lipopolysaccharide-LPS), TLR5 (flagellin-Flag), TLR7/8 (R848), TLR9 (CpG), NOD2 (muramyl dipeptide-MDP), proinflammatory cytokines interleukin 1 beta (IL-1β) and tumour necrosis factor (TNF) and the anti-inflammatory cytokine interleukin 10 (IL-10). Supernatant was collected after six and 20 h of stimulation. Following the pilot study, a stimulation assay was conducted in PBMCs from 40 individuals. Ligands yielding less than 200 pg/mL CCL20 in the pilot study were excluded (except for IL-10 as it is anti-inflammatory). PRR ligands and cytokines used for stimulation were MDP and Flagellin (both at 100 ng/mL) from Invivogen (San Diego, CA, USA) P3C (300 ng/mL) from EMC (Tuebingen, Germany), CpG (10 μM) from TibMolBiol (Berlin, Germany), IL-1β, IL-10 and TNF, all at 100 ng/mL from PeproTech (Rocky Hill, NJ, USA). Supernatant was collected after 20 h of stimulation. To determine whether CCL20 release from PBMCs occurred mainly secondarily to IL-1β, IL-1β neutralization assays were performed in PBMCs from three healthy donors. Cells were treated with anti-hIL-1β-IgG (cat.nr mabg-hil1b-3) or Mouse IgG1 isotype control (cat.nr mabg1-ctrlm) from Invivogen (San Diego, CA, USA) (1 μg/mL) for one hour prior to stimulation for six h with P3C (300 ng/mL), MDP (100 ng/mL) and IL-1β (100 ng/mL). Further, we performed CCL20 stimulation in PBMCs to investigate if CCL20 has additional functions to chemotaxis and antibacterial traits. In a pilot experiment, PBMCs from four healthy individuals were stimulated with CCL20 alone or in combination with TNF. Multiplex analysis covering 27 cytokines (Bio-Plex Pro, Human cytokine 27-plex Assay, Bio-Rad, Hercules, CA, USA) was used to screen for effects. Based on the pilot assay, PBMCs from 40 subjects were stimulated for 20 h with CCL20 (100 ng/mL) (R&D systems, Abington, UK) alone or in combination with TNF (100 ng/mL). To assess interindividual variability, we conducted the same CCL20 stimulation assay on PBMCs collected from three healthy controls. PBMCs from three healthy individuals were isolated from buffy coat and stored in multiple aliquots on liquid nitrogen. Three separate stimulation assays were conducted on frozen aliquots of PBMCs from all three donors. PBMCs from each individual were thawed and seeded with supplemented RPMI and stimulated following the same protocol as described above.

### 4.7. Measurements in Supernatant

Supernatant from the pilot assay of CCL20 stimulation in PBMCs was analysed by a multiplex assay, according to the manufacturer’s instructions. Supernatants from PBMC stimulation assays were thawed on ice and analysed by enzyme-linked immunosorbent assay (ELISA) for CCL20 (cat.nr DY360), IL-1β (cat.nr DY201) or IL-6 (DY206), using human Duo-Sets from R & D systems (Abingdon, UK), according to the manufacturer’s instructions.

### 4.8. Statistical Analysis

Data from stimulation assays were analysed using one-way ANOVA with Tukey’s multiple comparison post-test, or Kruskal-Wallis nonparametric test with Dunn’s multiple comparisons post-test if data was not normally distributed. Mann-Whitney *U*-test or unpaired *t*-test was used to detect group differences. Wilcoxon paired signed rank test and Friedman test with Dunn’s multiple comparisons post-test was used to compare effect of stimuli. Chi Square and Fisher test were used to assess differences in distribution of sex and medication between the groups in the sample sets. Statistics were performed in GraphPad Prism 7.0 (GraphPad Software Inc., San Diego, CA, USA).

### 4.9. Data Availability

The datasets generated during and/or analysed during the current study are available from the corresponding author on reasonable request.

## Figures and Tables

**Table 1:** Characteristics of subjects included in peripheral blood mononuclear cells (PBMC) analysis.

Characteristics	N	UC	CD
Number	8	16	16
Female sex	5	8	7
Age mean (range)	43 (22–68)	45 (19–76)	40 (24–57)
5-ASA/S-ASA	0	13	4
Corticosteroids	0	1	1
Mayo/HBI < 1	-	10	9
Mayo/HBI ≥ 1	-	6	7

Subjects’ sex, age and use of 5-ASA/S-ASA and corticosteroids are given in numbers. N = Healthy controls, UC = ulcerative colitis, CD = Crohn’s disease, 5-ASA/S-ASA = 5-aminosalicylic acid/sulphasalazine. HBI = Harvey-Bradshaw Index.

## Supplementary Materials

Supplementary materials can be found at: http://www.mdpi.com/1422-0067/19/10/3257/s1.

## Abbreviations

5-ASA	5-aminosalicylic acid
S-ASA	sulphasalazine
CCL20	C–C motif chemokine ligand 20
CCR6	C–C motif chemokine receptor 6
CD	Crohn’s disease
ELISA	enzyme-linked immunosorbent assay
hsCRP	High sensitivity C-reactive protein
IBD	inflammatory bowel disease
IL-1β	interleukin 1β
IL-10	interleukin 10
mRNA	messenger ribonucleic acid
MDP	muramyl dipeptide
P3C	pam3CysSK4
PRR	pattern recognition receptor
PBMCs	Peripheral blood mononuclear cells
qRT-PCR	quantitative real time polymerase chain reaction
TLR	toll-like receptor
TNF	Tumour necrosis factor
UC	ulcerative colitis
