# Supplementary material for: C-C Motif Ligand 20 (CCL20) and C-C Motif Chemokine Receptor 6 (CCR6) in Human Peripheral Blood Mononuclear Cells: Dysregulated in Ulcerative Colitis and a Potential Role for CCL20 in IL-1β Release
Source: Int J Mol Sci. 2018 Oct 20;19(10):3257. doi: 10.3390/ijms19103257 (PMC6214005; doi:10.3390/ijms19103257)
Supplement: Supplementary file 1 [file ijms-19-03257-s001.zip › ijms-350838-supplementary final version/ijms-350838 figure supplementary.pdf]

Supplementary materials:

Figure S1: CCL20 release in pilot stimulation assay

Figure S2: CCL20 and IL-6 release from PBMCs after IL-1β neutralization

Figure S3: CCL20 release from freshly isolated PBMCs

Figure S4: CCL20 mRNA in PBMCs

Figure S5: Gating strategies for CCR6 expressing PBMCs

Figure S6: Main cell populations in PBMCs

Table S1: Main lymphocyte and monocyte populations with CCR6⁺ cells

Data file 1: Flow cytometry data PBMCs

Data file 2: mRNA expression data CCR6 and CCL20 PBMCs

Data file 3: IL-1b release from PBMCs

Data file 4: CCL20 release from PBMCs

Data file 5: Correlations

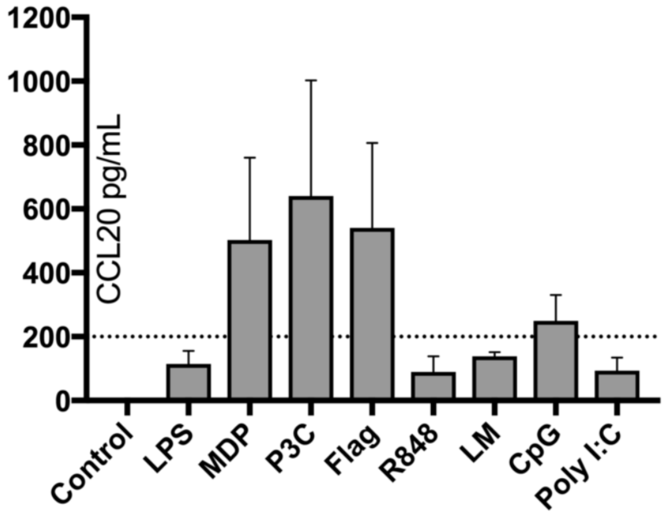

Figure S1. CCL20 release in pilot stimulation assay. PBMC from four healthy individuals were stimulated with ligands to toll-like receptor (TLR) 2/1 (pam3cysSK4 - P3C), TLR2/6 (lipomannan - LM), TLR3 (Poly IC), TLR4 (lipopolysaccharide - LPS), TLR5 (flagellin - Flag), TLR7/8 (R848), TLR9 (CpG) and NOD2 (muramyl dipeptide - MDP). Mean with SEM is shown. We chose a cut-off of mean CCL20 release into supernatant at 200pg/mL (dotted line), which was used to determine ligands in the 40 subjects stimulation assay.

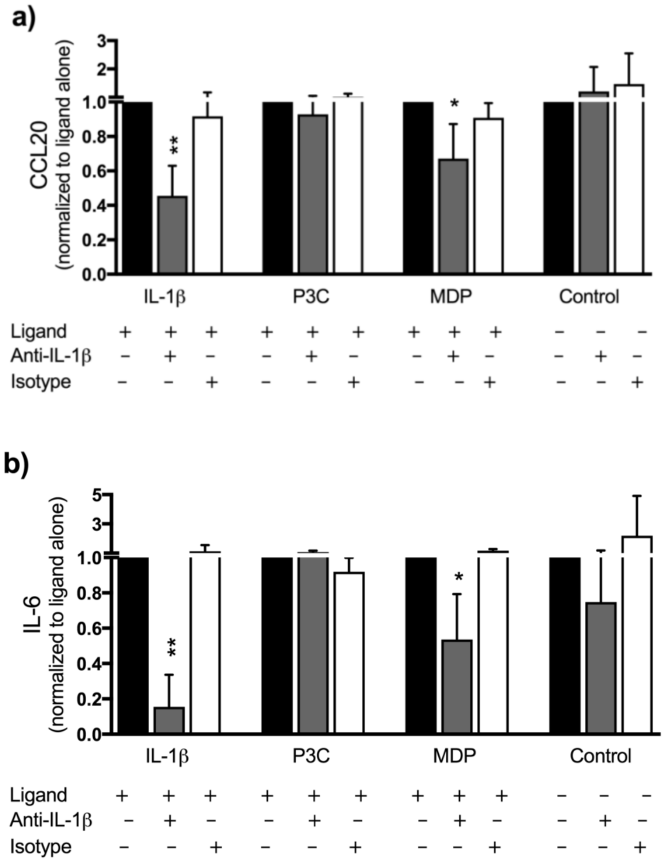

Figure S2. CCL20 and IL-6 release from PBMCs after IL-1β neutralization Effect of IL-1β neutralization during lipopeptide Pam3CysSK4 (P3C) and muramyl dipeptide (MDP) stimulation in PBMCs from three healthy controls. PBMCs were pre-treated with neutralizing antibody to interleukin 1β (IL-1β) (with anti-hIL-1β-IgG) 1 g/mL or isotype control (mouse IgG1) prior to stimulation with ligands P3C (300ng/mL), MDP (100ng/mL) or IL-1β (100ng/mL). Mean with SEM of three assays is plotted, normalized to stimulation with ligand. Statistical comparison was performed using unpaired t-test with levels of significance denoted by ** $p<0.01$ versus ligand. * $p<0.05$ versus ligand. **a)** CCL20 release. **b)** IL-6 release.

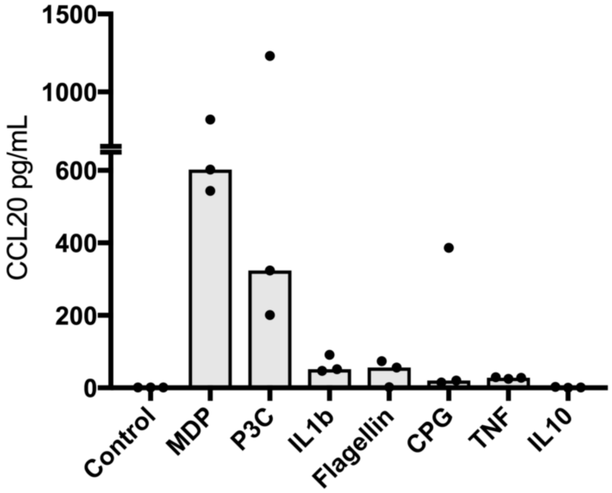

Figure S3. CCL20 release from freshly isolated PBMCs. CCL20 (pg/mL) release from freshly isolated PBMCs (n=3), following stimulation with lipopeptide Pam3CysSK4 (P3C) (TLR1/2), peptidoglycan component muramyl dipeptide (MDP) (NOD2), unmethylated CpG dinucleotides (CpG) (TLR9), flagellin (TLR5), interleukin (IL) 1β (IL-1β), IL-10, and tumour necrosis factor (TNF). Individual values and median are plotted.

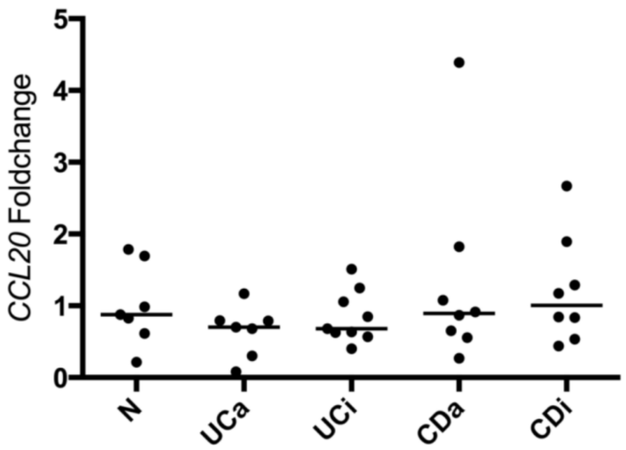

Figure S4 CCL20 mRNA in PBMCs. CCL20 mRNA level in PBMCs from 38 subjects, in healthy controls (N)(n=7), active ulcerative colitis (UCa)(n=7) inactive ulcerative colitis (UCi)(n=8), active Crohn's disease (CDa)(n=8) and inactive Crohn's disease (CDi)(n=8). Foldchanges were determined relative to the reference genes *beta actin*, *TATA binding protein* and *eukaryote 18s rRNA*, and to the mean expression level of healthy control individuals (N). Individual fold changes and median are plotted.

Sup. Fig. 5

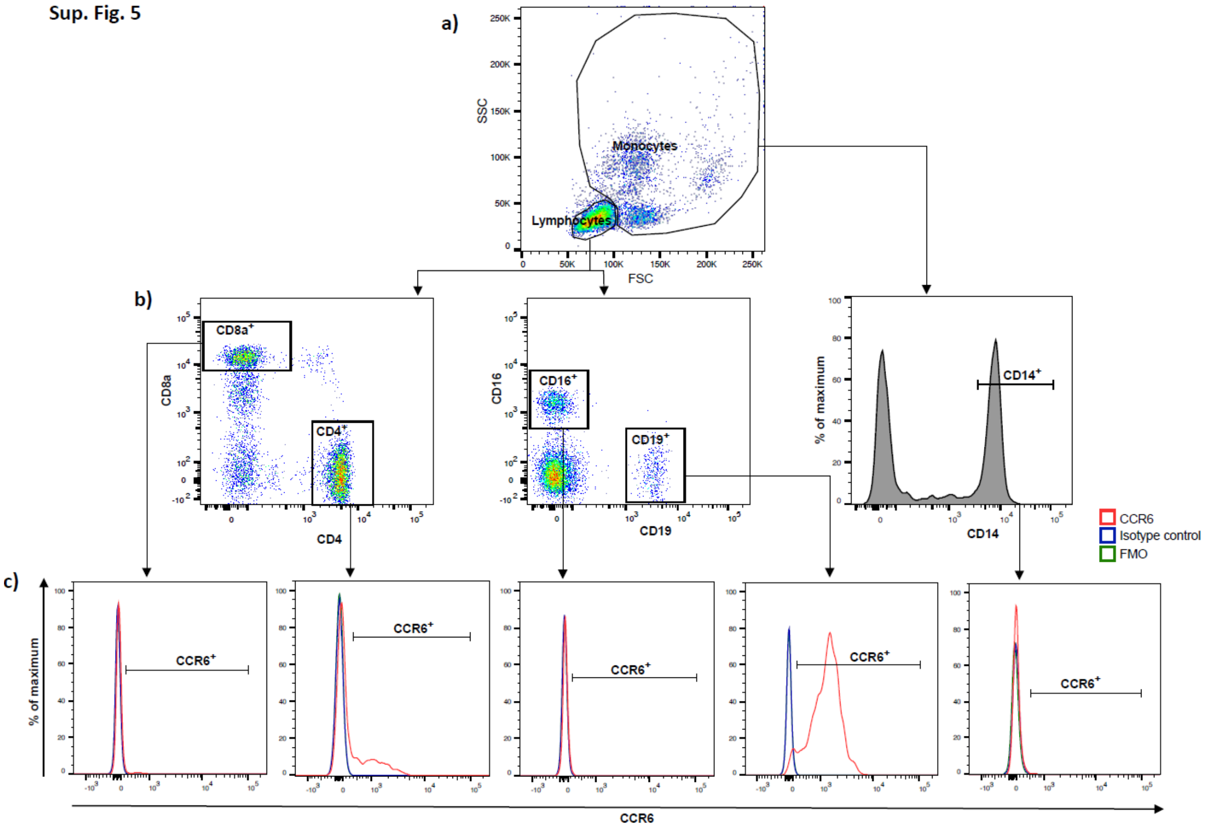

Figure S5 Gating strategies for CCR6 expressing PBMCs. Representative flow cytometry plots from one healthy control using six-colour staining of PBMC, counting 10.000 events. **a)** FSC/SSC-dot plot of lymphocyte and monocyte cell population. **b)** Dot plots of the defined lymphocyte population expressing CD4, CD8, CD16 and CD19, and histogram of CD14 expressing monocyte population. **c)** IgG isotype control (blue) and FMO (fluorescence minus one) (green) was used for gating of CCR6 expression within each lymphocyte and monocyte subpopulation.

\

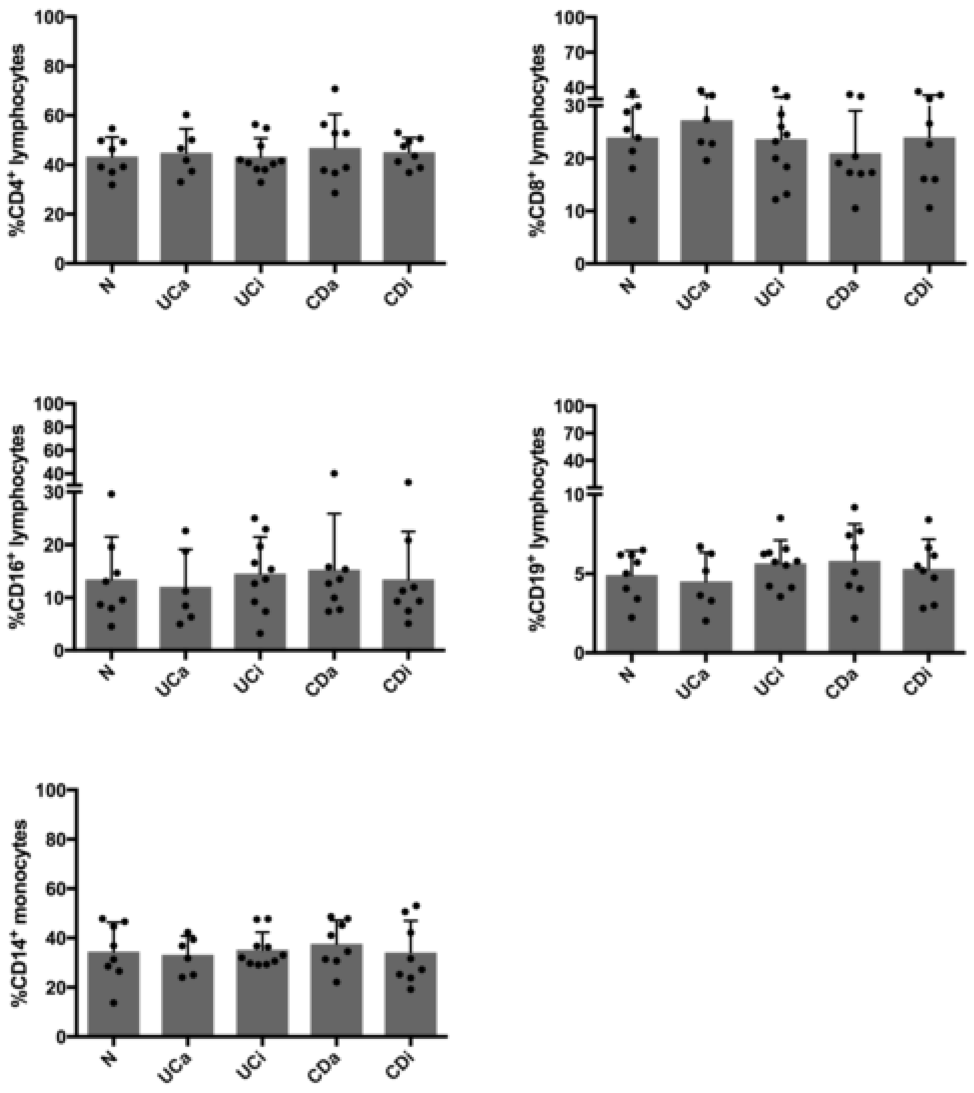

Figure S6 Main cell populations in PBMCs. Main cell population ($CD4^+$ T cells, $CD8^+$ T cells, $CD16^+$ NK cells, $CD19^+$ B cells, and $CD14^+$ monocytes,) in 1million PBMCs from 40 subjects. Results in healthy controls (N)(n=8), active ulcerative colitis (UCa)(n=6) inactive ulcerative colitis (UCi)(n=10), active Crohn's disease (CDa)(n=8) and inactive Crohn's disease (CDi)(n=8) are plotted. Bars show mean frequency (%) and SD, and individual values are plotted.
