# Supplementary material for: C-C Motif Ligand 20 (CCL20) and C-C Motif Chemokine Receptor 6 (CCR6) in Human Peripheral Blood Mononuclear Cells: Dysregulated in Ulcerative Colitis and a Potential Role for CCL20 in IL-1β Release
Source: Int J Mol Sci. 2018 Oct 20;19(10):3257. doi: 10.3390/ijms19103257 (PMC6214005; doi:10.3390/ijms19103257)
Supplement: Supplementary file 1 [file ijms-19-03257-s001.zip › ijms-350838-supplementary final version/ijms-350838-Table Supplementary Materials.pdf]

Table S1 Main Lymphocyte and Monocyte Populations with CCR6⁺ Cells.

		N	UCa	UCi	CDa	CDi	Sign.
**Number of subjects**		8	6	10	8	8	-
**CD4⁺**	**FOP**	43±7.8	45±9.7	43±7.5	47±13.7	45±5.9	n.s
	**MFI**	4577±490	4565±599	4683±451	4551±552	4435±310	n.s
**CD8a⁺**	**FOP**	24±8.4	27±6.8	24±8.2	21±8.0	24±9.2	n.s
	**MFI**	13906±1040	13571±1513	14070±1265	14000±960	13967±1472	n.s
**CD14⁺**	**FOP**	35±11.8	33±7.6	35±7.1	38±9.5	34±12.8	n.s
	**MFI**	7293±584	7239±1301	7826±659	7581±621	7495±283	n.s
**CD16⁺**	**FOP**	14±8.0	12±7.1	15±6.9	15±10.5	14±9.0	n.s
	**MFI**	2551±600	2638±533	2125±595	2329±716	2285±652	n.s
**CD19⁺**	**FOP**	4.9±1.5	4.5±1.8	5.7±1.4	5.8±2.3	5.3±1.8	n.s
	**MFI**	3710±319	3600±278	3777±331	3683±453	3704±593	n.s
**CCR6⁺CD4⁺**	**FOP**	13.1±8.5	7.4±4.4	14.6±6.8	10.5±7.4	8.2±4.3	UCa vs UCi p=0.038
	**MFI**	748±185	697±143	793±185	722±151	676±122	n.s
**CCR6⁺CD8⁺**	**FOP**	4.2±3.9	1.4±0.8	2.8±1.9	4.5±3.7	2.7±1.4	UCa vs N p=0.043<br>UCa vs CDa p=0.029
	**MFI**	429±106	379±195	447±125	391±79	423±122	n.s
**CCR6⁺CD14⁺**	**FOP**	1.0±0.9	1.0±0.5	0.8±0.4	1.0±0.5	0.8±0.5	n.s
	**MFI**	789±321	795±181	1106±846	831±353	710±172	n.s
**CCR6⁺CD16⁺**	**FOP**	0.4±0.4	0.3±0.2	0.4±0.5	0.5±0.4	0.3±0.2	n.s
	**MFI**	325±173	431±291	471±354	682±986	432±429	n.s
**CCR6⁺CD19⁺**	**FOP**	90±6.0	87±11.3	92±8.8	91±8.5	86±16.5	n.s
	**MFI**	1023±301	1072±429	1173±326	1058±364	961±327	n.s

CCR6⁺ population within CD4⁺ lymphocytes, CD8⁺ lymphocytes, CD16⁺ NK cells, CD19⁺ lymphocytes, and CD14⁺ monocytes. Frequency of parental (FOP) and Mean Fluorescent Intensity (MFI) is given in mean with standard deviation in the disease groups healthy controls (N), active ulcerative colitis (UCa), active Crohn's disease (CDa), inactive UC (UCi) and inactive CD (CDi). n.s: not significant.
